# Curcumin Supplementation Decreases Intestinal Adiposity Accumulation, Serum Cholesterol Alterations, and Oxidative Stress in Ovariectomized Rats

**DOI:** 10.1155/2016/5719291

**Published:** 2015-11-23

**Authors:** Maurilio da Silva Morrone, Carlos Eduardo Schnorr, Guilherme Antônio Behr, Juciano Gasparotto, Rafael Calixto Bortolin, Katia da Boit Martinello, Bernardo Saldanha Henkin, Thallita Kelly Rabello, Alfeu Zanotto-Filho, Daniel Pens Gelain, José Cláudio Fonseca Moreira

**Affiliations:** Departamento de Bioquímica, Centro de Estudos em Estresse Oxidativo, Universidade Federal do Rio Grande do Sul (UFRGS), Laboratório 32, Anexo Dpto de Bioquímica, Bairro Santana, Rua Ramiro Barcelos 2600, 90035-003 Porto Alegre, RS, Brazil

## Abstract

The aim of this study was to investigate the potential of curcumin oral supplementation (50 and 100 mg/Kg/day, for 30 days) in circumventing menopause-associated oxidative stress and lipid profile dysfunctions in a rat ovariectomy (OVX) model. Female Wistar rats were operated and randomly divided into either sham-operated or OVX groups. Sham-operated group (*n* = 8) and one OVX group (*n* = 11) were treated with vehicle (refined olive oil), and the other two OVX groups received curcumin at 50 or 100 mg/Kg/day doses (*n* = 8/group). OVX vehicle-treated animals presented a higher deposition of intestinal adipose tissue as well as increased serum levels of IL-6, LDL, and total cholesterol when compared to sham-operated rats. In addition, several oxidative stress markers in serum, blood, and liver (such as TBARS, carbonyl, reduced-sulphydryl, and nonenzymatic antioxidant defenses) were altered toward a prooxidant status by OVX. Interestingly, curcumin supplementation attenuated most of these parameters to sham comparable values. Thus, the herein presented results show that curcumin may be useful to ameliorate lipid metabolism alterations and oxidative damage associated with hormone deprivation in menopause.

## 1. Introduction

Ageing is accompanied by changes in the activity of several genes involved in the control of metabolism, antioxidant systems, DNA repair, cellular senescence, and death [1]. Even though human lifespan in the 21th century has increased all over the world, especially in developed countries, the age when women enter their major age-related hormonal change (i.e., menopause) has remained constant, at around 50 years [2]. It means this phase could take part in almost one-third of women's life. In fact, menopause is characterized by loss of ovarian function and subsequent decrease in serum levels of estrogen and progesterone, which are associated with the development/acceleration of arteriosclerosis, skin aging, and immune dysfunctions among others [2].

Oxidative stress has been defined as an unbalance between increased reactive oxygen species (ROS) production and a noncorrespondent enzymatic and nonenzymatic antioxidant activity [3, 4]. Several evidences have described menopause as a prooxidant and inflammatory state, which directly impact the development of several ageing and oxidative stress-associated diseases [2]. For example, menopause is a risk factor associated with the onset or/and progression of cardiovascular diseases [5, 6], and much of this correlation is attributed to the benefic effects of female sexual hormones in protecting the cardiovascular system, by either acting as inducers of antioxidant genes or functioning as endogenous free-radicals scavengers* per se* [7, 8]. Because the oxidative stress consequent of the lack sexual hormones has been a major concern in the menopause and postmenopausal landscapes [9, 10], hormone replacement therapy (HRT) has been chosen as the standard approach to alleviate menopause-associated symptoms, thus preventing the clinical consequences of an estrogen-deficient state. However, because of the possible negative effects associated with long-term HRT, especially the increased risk of thromboembolic accidents, stroke, and breast cancer [2, 11], HRT has lost ground among women, and a growing interest in alternative strategies has been established. In this regard, there is a particular interest in validating the antimenopausal properties of natural herbs with antioxidant/anti-inflammatory potential as well as few or even none significant side effects.

Curcumin has been described to regulate signaling and metabolic pathways by modulating diverse molecular events, including transcription factors activity, cytokines production, and antioxidant status, as well as cell proliferation and apoptosis genes [12–14]. As an antioxidant, for example, curcumin is able to prevent the drop in hepatic glutathione and decreases lipid peroxidation in the hepatocarcinogenesis promoted by N-nitrosodiethylamine in male Wistar rats [15]. In ovariectomized (OVX) rodent, which is a classical animal model of chronic progressive bone loss/osteoporosis, curcumin supplementation exhibited bone sparing effects [16–18]. Besides osteoporosis, curcumin could also act beneficially on other symptoms caused by menopause such as arteriosclerosis, obesity, and other pathologies associated with oxidative stress progression along this period [19]. In this context, even though not only have studies on the efficacy and safety of curcumin been claimed, but also ambiguous results and a complete lack of reports from human clinical trials show that the potential use of curcumin for prophylaxis or treatment of postmenopausal remains underestimated [11].

Considering the aforementioned, the aim of this study was to investigate the effects of curcumin supplementation (50 and 100 mg/Kg/day, during 30 days) on adiposity accumulation and serum biochemical and oxidative stress parameters in a menopause model of ovariectomized (OVX) Wistar rats, aiming to determine its potential to attenuate menopause-associated metabolic and oxidative alterations caused by hormone deprivation.

## 2. Material and Methods

All experimental procedures were performed in accordance with the National Institutes of Health Guide for Care and Use of Laboratory Animals (NIH Publication Number 80–23 revised 1996) and were carried out according to the determinations of the Brazilian Council for the Control of Animal Experimentation (CONCEA). Experiments were approved by the University Animal Research Ethic Committee (Register Number 25320).

### 2.1. Animals and Reagents

Female Wistar rats (200–250 g) in regular estrous cycles were obtained from our breeding colony. The animals were maintained in groups of five individuals with access to standard pellet food and water* ad libitum.* The animals were maintained under a 12-hour light–dark cycle (7 am–7 pm) in a temperature-controlled room (23 ± 1°C). Curcumin was purchased from Sigma Chemical Co. (St. Louis, MO, USA). Ketamine hydrochloride was from Virbac Ltda (Jurubatuba, SP, Brazil) and xylazine hydrochloride was from Vetbrands Ltda (Goiania, GO, Brazil). All other chemicals used in the study were purchased from Sigma Chemical Co. (St. Louis, MO, USA).

### 2.2. Surgical Procedures

The rats were allowed 2 weeks to acclimatize before the beginning of the experimental protocol. Afterwards, seventy-day-old female rats were randomly divided into either sham-operated group (*n* = 8) or OVX group (*n* = 27). Rats were anesthetized by intraperitoneal injection of ketamine (100 mg/kg) plus xylazine (15 mg/kg), and ovariectomy was performed under aseptic conditions as previously described [20].

### 2.3. Treatment

Two months after surgery, the animals were treated with curcumin or vehicle every other day for a total of 30 days. All treatments were carried out at night. Sham-operated (*n* = 8) and one OVX group (*n* = 11) were treated with vehicle (refined olive oil) and the other two OVX groups (*n* = 8 each) received curcumin at 50 and 100 mg/Kg/day doses, respectively. Treatment was performed via intragastric gavage in a maximum volume of 0.4 mL. The animals were weighted weekly.

### 2.4. Sample Acquisition

After 30 d treatment (90 d after ovariectomy), the rats were decapitated and blood, serum, liver, visceral adipose tissue, and uterus samples were collected for analysis. The uterus was cut above the cervical junction, the visible fat was removed, and the cleaned uterus was weighed. Intestinal adipose tissue (IAT) was carefully removed from small intestine and weighted. The ratios of the uterus and IAT weight relative to animal weight were calculated. Whole blood was collected and serum was separated. Blood, serum, and liver samples were stored at −80°C for subsequent analyses. Blood samples were frozen and thawed (−80°C/25°C) twice and centrifuged (900 g, 5 min) to remove debris. The liver samples were dissected and frozen at −80°C·s.

### 2.5. Serum Markers Profiling

Serum samples were used to determine the levels of high-density lipoprotein (HDL), low-density lipoprotein (LDL), very low-density lipoprotein (VLDL), total cholesterol, and total triglycerides, as well as the hepatotoxicity markers aspartate aminotransferase (AST) and alanine aminotransferase (ALT) activities. All these parameters were assayed using commercial kits (Labtest Diagnóstica SA; Lagoa Santa, MG, Brazil). Serum interleukin-6 (IL-6) levels were determined by ELISA following manufacturer's instructions (Catalog Number RAB0311, Sigma Aldrich, USA).

### 2.6. Redox Profile in Blood, Serum, and Liver Samples

#### 2.6.1. Antioxidant Enzymes Activity Quantification

Superoxide dismutase activity was estimated from the inhibition of superoxide anion-dependent adrenaline autooxidation in a spectrophotometer at 480 nm as previously described [21]. Results were expressed as units of SOD/mg protein. Catalase activity was assayed by measuring the ratio of decrease in hydrogen peroxide (H_2_O_2_) absorbance in a spectrophotometer at 240 nm [22]. To determine GPx activity, the rate of NAD(P)H oxidation was measured in a spectrophotometer at 340 nm in the presence of reduced glutathione,* tert*-butyl hydroperoxide, and glutathione reductase as previously described [23].

#### 2.6.2. Nonenzymatic Antioxidant Potential (TRAP Assay)

We used the total reactive antioxidant potential test (TRAP) as an index of tissue nonenzymatic antioxidant potential. This assay is based on the decrease of chemiluminescence produced from the reaction of 2,20-azobis[2-amidinopropane] (AAPH) derived peroxyl radical with luminol due to free radical quenching by the antioxidant compounds present in the sample [24]. Briefly, we prepared AAPH solutions and added luminol (“System” solution, 100% chemiluminescence); thereafter, we waited 2 h for the system to stabilize before performing the first reading. After the addition of the samples, the chemiluminescence was monitored over a 40 min period, the results were transformed to percentages, and the area under curve (AUC) was calculated as previously described [25]. The samples displaying lower AUC will be those with higher antioxidant capacity.

#### 2.6.3. Oxidative Damage Markers

The formation of thiobarbituric acid reactive species (TBARS) was quantified as an index of lipid peroxidation as previously described [26]. The samples were mixed with 0.6 mL of 10% trichloroacetic acid (TCA) and 0.5 mL of 0.67% thiobarbituric acid and then heated in a boiling water bath for 30 min. TBARS were determined at 532 nm in a spectrophotometer reader. Results are expressed as *η*Mol of TBARS/mg protein.

Oxidative damage to proteins was measured by quantification of carbonyl groups as previously described [27]. This method is based on the reaction of dinitrophenylhydrazine (DNPH) with protein carbonyl groups, and the absorbance was read in a spectrophotometer at 370 nm. Results are expressed in *μ*mol carbonyls/mg protein.

In order to measure the levels of reduced thiol (-SH) groups in protein and nonprotein fractions from rat tissues, we used the Ellman's reagent based assay [28]. For total SH content measurement, a 50–100 *μ*g sample aliquot was diluted in PBS and reacted with 10 mM 5,5-dithionitrobis 2-nitrobenzoic acid. After 60 min incubation at room temperature, the absorbance was read in a spectrophotometer set at 412 nm. To assess the nonprotein SH content (which includes glutathione and other small peptides), 1 mg protein aliquot was reacted with trichloroacetic acid (10% v/v) for deproteinization and centrifuged (10,000 g/10 min), and the supernatants were used to measure the level of SH in the protein-free fraction. Results are expressed as “mmol SH groups/mg protein” or “*μ*mol SH groups/mg protein” for protein and nonprotein SH, respectively.

### 2.7. Statistical Analysis

Data were expressed as average ± standard error (SEM). Differences were compared by one-way ANOVA, followed by Tukey's test. *P* < 0.05 were considered significant.

## 3. Results

### 3.1. Body Weight Gain, Uterine Tissue, and Intestinal Adipose Tissue Ratio

Table 1 shows the body weight gain at the end of a 30-day treatment period. Irrespective of the curcumin supplementation, weight gain (g) was significantly higher in all the OVX groups when compared to sham. We also collected, weighted, and analyzed the uterine morphology at the end of the treatments (Table 1). We observed that sham animals presented different uterine morphology according to the estrous cycle phase. Two sham rats presented characteristic proestrus (high fluid content and thick tissue) whereas five others displayed nonproestrus, estrus, or diestrus morphologies. All OVX rats, independent of the treatment, showed a significant reduction in uterine tissue weight when compared to sham groups. Uterus from OVX rats was found highly atrophied, confirming the absence of ovarian hormones secretion and ovulation along this period. Intestinal adipose tissue (IAT) was also removed and weighted. OVX vehicle-treated animals presented a higher IAT/body weight ratio when compared to sham rats and supplementation with curcumin decreased IAT accumulation (Table 1).

### 3.2. Effects of Curcumin on IL-6, Serum Lipid Profile, and Tissue Damage Biomarkers in OVX Rats

It has been established that the abdominal/visceral adiposity accumulation in menopause sets out predisposition to a proinflammatory status due to macrophages recruitment and activation with the adipose tissue and/or imbalance between leptin and adiponectin productions by adipocytes [29, 30]. In our model, OVX promoted slight but significant increases in serum levels of IL-6, which were restored to control levels by curcumin at both concentrations tested (Table 2).

Taking into account the positive effect of curcumin on IAT accumulation, we sought to investigate whether curcumin could alter serum lipid profiles in the OVX model (Table 2). We detected that basal levels of LDL and total cholesterol increased in OVX compared to sham-operated rats. Animals that received curcumin showed LDL and total cholesterol levels comparable to to sham values. Even though the OVX model did not change HDL, VLDL, and triglycerides concentrations, at least at the end of the 90 days studied, curcumin supplementation decreased the basal levels of these circulating lipids. Besides lipid markers, AST and ALT serum activities were quantified in order to address the impact of OVX and curcumin upon systemic tissue damage. In all OVX groups, AST activity presented a modest but significant increase when compared to sham, and curcumin was not able to restore AST to sham levels. ALT activities did not differ among all groups investigated (Table 2).

### 3.3. Effects of Curcumin on the Antioxidant Profile in OVX Rats

**Figure 1:**
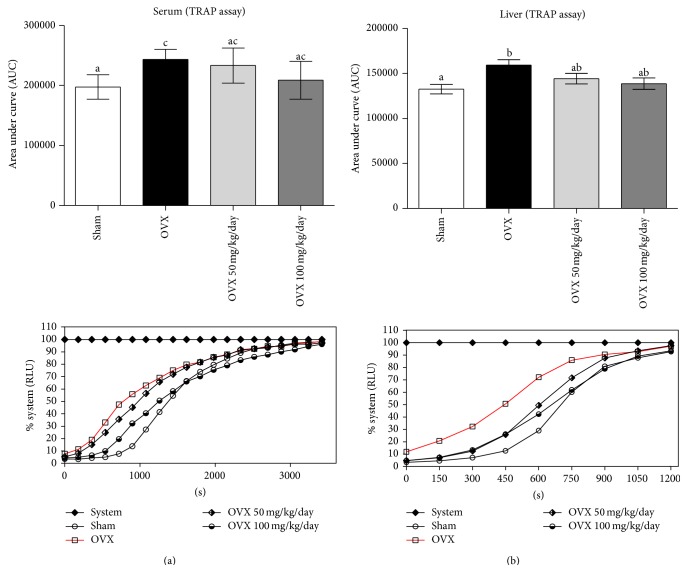
Effects of ovariectomy and curcumin supplementation on serum (a) and liver (b) nonenzymatic antioxidant potential. An experiment's representative graphic and the area under curve of total reactive antioxidant potential were analyzed on both samples. Data are expressed by mean ± SEM (sham *n* = 8, OVX *n* = 11, OVX 50 *n* = 8, and OVX 100 *n* = 8) and the experiments were performed in triplicate. Statistical difference from sham group: ^b^ *P* < 0.05, ^c^ *P* < 0.01 (one-way ANOVA followed by the post hoc Tukey's test).

Switching cellular metabolism toward a prooxidant environment is a hallmark of menopause. Substances such as reduced-sulphydryl groups (R-SH), vitamins A and E, albumin, GSH, and polyphenols among others possess chemical characteristics that directly affect the antioxidant balance in serum and tissues. In OVX rats, we observed a decrease in the nonenzymatic free radical scavenger potential, which was detected in both serum (OVX = 244456 ± 6572, sham = 197410 ± 7209 AUC units) and liver (OVX = 161070 ± 6808, sham = 132628 ± 5132 AUC units) as assessed by TRAP assay (Figure 1). Curcumin treatment made OVX animals enriched in nonenzymatic antioxidants as determined from AUC in serum (OVX 50 = 233252 ± 11902, OVX 100 = 208913 ± 12835) and liver (OVX 50 = 144215 ± 5854, OVX 100 = 138784 ± 6299) and also clearly observed from the determination of “time for 50% induction” of chemiluminescence (Table 3).

**Figure 2:**
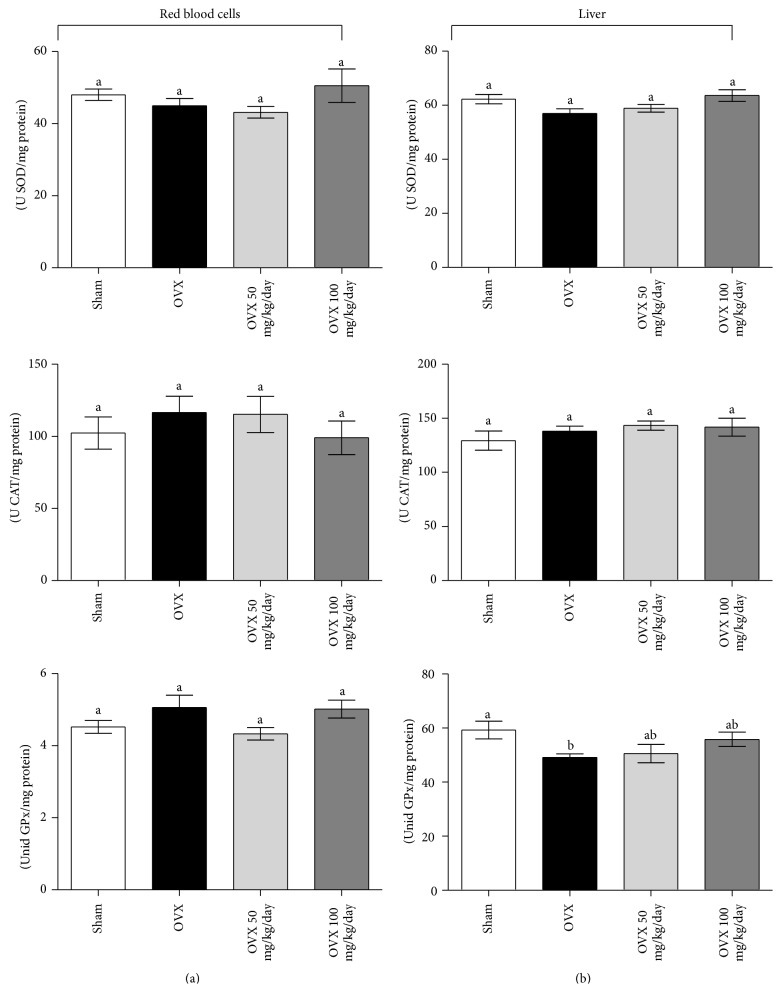
Effects of ovariectomy and curcumin supplementation on blood and liver antioxidant enzyme activities. Total superoxide dismutase (SOD) activity, catalase (CAT) activity, and glutathione peroxidase (GPx) activity were measured in blood (a) and liver (b) samples. Data are mean ± SEM (sham *n* = 8, OVX *n* = 11, OVX 50 *n* = 8, and OVX 100 *n* = 8) and the experiments were performed in triplicate. Statistical difference from sham group: ^b^ *P* < 0.05 (one-way ANOVA followed by the post hoc Tukey's test).

Besides the aforementioned nonenzymatic antioxidants, we quantified the activity of the main antioxidant enzymes (Figure 2). Different from the effect on serum nonenzymatic defenses, neither OVX nor curcumin treatment caused modifications in antioxidant enzymes activities (as units/mg protein) in red blood cells homogenates. All OVX groups presented similar activities of SOD (OVX = 44.9 ± 2.0, OVX 50 = 43.2 ± 1.6, and OVX 100 = 50.5 ± 4.7), CAT (OVX = 116.5 ± 11.4, OVX 50 = 115.2 ± 12.5, and OVX 100 = 99.0 ± 11.7), and GPx (OVX = 5.1 ± 0.3, OVX 50 = 4.3 ± 0.17, and OVX 100 = 5.0 ± 0.25) when compared to sham (sham_SOD_ = 48.0 ± 1.6, sham_CAT_ = 102.3 ± 11.2, and sham_GPx_ = 4.5 ± 0.18).

In liver, GPx activity decreased in OVX samples when compared to sham-operated group (OVX = 50.0 ± 1.6, sham = 59.3 ± 3.3; *P* < 0.05), and curcumin was able to prevent the decrease in GPx at both of the doses tested herein (OVX 50 = 50.5 ± 3.4, OVX 100 = 55.8 ± 2.7) (Figure 2(b)). SOD (sham = 62.3 ± 4.5, OVX = 53.9 ± 4.9, OVX 50 = 58.9 ± 4.1, and OVX 100 = 63.6 ± 6.1) and CAT (sham = 129.4 ± 23.3, OVX = 138.0 ± 15.3, OVX 50 = 143.3 ± 11.9, and OVX 100 = 141.8 ± 23.4) activities across OVX groups also did not alter.

### 3.4. Oxidative Damage Markers and SH Status in Blood, Liver, and Serum

**Figure 3:**
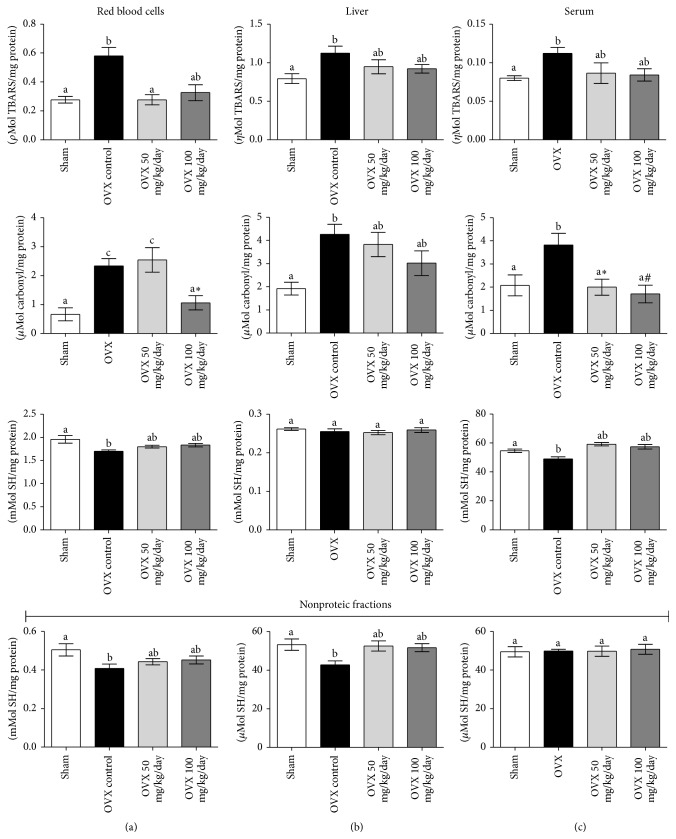
Effects of ovariectomy and curcumin supplementation on blood, liver, and serum thiol reduced content and oxidative damage markers. Proteic and nonproteic SH content, protein carbonyls groups, and lipid peroxidation were analyzed in blood (a), liver (b), and serum (c) samples. Data are expressed by mean ± SEM (sham *n* = 8, OVX *n* = 11, OVX 50 *n* = 8, and OVX 100 *n* = 8) and the experiments were performed in triplicate. Statistical difference from sham group: ^b^ *P* < 0.05, ^c^ *P* < 0.01. Statistical difference from OVX group: ^*∗*^ *P* < 0.05, ^#^ *P* < 0.01 (one-way ANOVA followed by the post hoc Tukey's test).

Damage to biomolecules is a major consequence of a prooxidant imbalance. We observed that OVX, and its consequent menopause-like changes, promoted a significant effect on the oxidative stress markers. TBARS (lipid peroxidation), carbonylated proteins (carbonyl index), and nonproteic and protein SH contents were altered by OVX in different tissues (Figure 3). TBARS levels (*ρ*mol/mg protein) and carbonylated proteins (*μ*mol/mg protein) increased in erythrocytes of OVX vehicle-treated animals (OVX = 0.48 ± 0.06, 2.3 ± 0.25, sham = 0.28 ± 0.02, 0.76 ± 0.32, resp.) while both nonproteic (*μ*mol/mg protein) and proteic (mmol/mg protein) SH contents decreased (OVX = 0.41 ± 0.02, 1.70 ± 0.03, sham = 0.50 ± 0.03, 1.95 ± 0.08; *P* < 0.05) when compared to sham (Figure 3(b)). These changes suggest a typical prooxidant status damaging both proteins and lipids in the blood of OVX animals. Curcumin supplementation attenuated the OVX-induced damage by decreasing lipoperoxidation (TBARS) and restoring of nonprotein and protein sulfhydryl homeostasis to levels comparable to sham. Higher doses (100 mg/Kg/day) of curcumin were also able to restore carbonylated proteins to sham levels in erythrocytes (Figure 3(b)).

As observed in red blood cells, oxidative damage profiling in liver samples revealed that OVX increased lipoperoxidation (TBARS) and proteins carbonylation (OVX = 1.12 ± 0.09, 4.26 ± 0.44, sham = 0.79 ± 0.06, 1.92 ± 0.27, resp.; *P* < 0.05) and 50 and/or 100 mg/Kg/day curcumin was able to prevent the oxidative damage to these biomolecules (Figure 3(c)). Protein SH groups did not change across the experimental groups. On the other hand, OVX depleted nonprotein sulfhydryl levels (OVX = 42.7 ± 2.1, sham = 53.2 ± 2.9 *μ*mol/mg protein; *P* < 0.05), which were restored by curcumin supplementation.

In serum, the antioxidant effects of curcumin were much similar to those observed in red blood cells and liver samples. OVX increased serum carbonyl groups when compared to sham (sham = 2.08 ± 0.45, OVX = 3.99 ± 0.61; *P* < 0.05), and treatment with curcumin rescued protein damage to sham levels (OVX + 50 = 2.00 ± 0.35, OVX + 100 = 1.71 ± 0.38) (Figure 3(a)). Protein sulphydryl content decreased in OVX animals (sham = 54.7 ± 1.2, OVX = 49.0 ± 1.4; *P* < 0.05), and curcumin supplementation restored it to sham levels (OVX 50 = 59.1 ± 1.1, OVX 100 = 57.4 ± 1.5; *P* < 0.05). On the other hand, neither OVX nor curcumin altered nonprotein SH content in the serum. Serum TBARS were increased by OVX, but curcumin was not able to statistically block this phenomenon. Hence, the aforementioned results show that curcumin intake optimizes or/and maintains the antioxidant status in OVX animals, thus protecting from oxidative stress-associated damage to biomolecules in different tissues. This effect seems to be more likely attributed to increments in the nonenzymatic potential than changes in the antioxidant enzymes activity, agreeing with previous studies from other models of curcumin supplementation [15, 31, 32].

## 4. Discussion

Menopause is characterized by a complete failure to produce progesterone and estrogen and, thus, represents a situation of premature aging. Sex hormone insufficiency is related to deep physiological alterations, which include increase in oxidative stress, bone loss, weight gain, and cardiovascular dysfunction among other complications associated with this period in animals and humans [33].

Menopause experimental models are widely used to reproduce the main aspects and changes associated with female hormone deprivation. The state-of-art procedure to mimic menopause in rodents is the bilateral ovariectomy. Not surprisingly, along the last years, there has been a great increase in the number of publications focusing on the metabolic alterations and menopause-related symptoms in OVX models, such as early aging of nervous and immune system [34], weight gain [35], behavioral changes [23], cardiovascular dysfunction [36], insulin resistance [37, 38], and alterations in cytokines levels (e.g., TNF-*α*, IL-1, IL-6, and IL-10) [39]. OVX rats also present increased oxidative stress levels and, consequently, an accelerated aging process in different tissues [7, 40, 41]. In this study, we observed that oral curcumin was able to prevent a number of biological impairments associated with hormone deprivation. Alterations in the levels of some lipid markers, IAT deposition, and, mainly, improvements in the antioxidant potential in blood and liver were observed after a 30-day supplementation, which is a noteworthy result given the well-recognized clinical safety of curcumin [42]. Given that most women are still reluctant to take the risks associated with HRT [2], diets based on antioxidants may help to protect menopausal and postmenopausal women against the high levels of oxygen stress implied in the acceleration of the arteriosclerotic process and skin aging, among others, that take place during middle age. However, taking into account that many menopausal and postmenopausal women actually do not consume the recommended five daily rations of such a healthy diet [43], they might obtain some benefit from dietary supplements. Here, we provided evidences that curcumin could enter as a possible alternative supplementation to alleviate the oxidative damage and lipid metabolism imbalances caused by sexual female hormone deprivation in menopause.

In fact, postmenopausal women showed higher levels of the prooxidant biomarkers MDA, 4-hydroxynonenal (4-HNE), and oxidized LDL, when compared to premenopausal subjects [44]. Much of the prooxidant condition associated with menopause is owed to the absence of estrogen, which, for example, has been reported to protect vascular smooth muscle cell membrane phospholipids against peroxidation [45] beyond preventing oxidative stress- induced endothelial cell apoptosis in rat models [46]. Previous studies underlie our results, since OVX rats display impaired total antioxidant capacity in serum and liver, which was accompanied by an increase in different oxidative damage markers [35, 47]. Our study revealed that neither ovariectomy nor curcumin had major effects upon the antioxidant enzymatic machinery. In the flip side, OVX depleted nonenzymatic antioxidants, which were restored by curcumin supplementation. Compounds like vitamins A, C, and E, uric acid, and sulphydryl reduced molecules as GSH accounts for most of the nonenzymatic antioxidants in biological systems. It is already shown by Dilek et al., 2010, [48] that OVX in rats decreases plasma concentrations of vitamins A, C, and E compared to healthy animals. We herein showed that depletion of nonenzymatic antioxidant by OVX was enough to result in free-radicals insult, which damaged different molecules in OVX rats. We demonstrated that curcumin supplementation could restore the nonenzymatic antioxidant buffer potential to protect biomolecules from damage in the context of female sexual hormone deprivation by OVX. Curcumin decreased the levels of carbonylated proteins and lipoperoxides in blood and liver compartments and also restored protein and nonprotein thiol homeostasis, without, overall, affecting enzymatic defenses in OVX rats. In a major perspective, it seems that the idea of supplementing antioxidants in menopause could be extended out of the curcumin context. For example, Abbas and Elsamanoudy [49] have observed increases in MDA levels and decreases in both enzymatic and nonenzymatic (GSH levels, GPx, CAT, and SOD activities) defenses in liver of OVX Sprague-Dawley rats, which were improved by vitamin E administration.

Obesity and increase in food intake are very common factors associated with menopause, leading to weight gain, gradual reduction in lean body mass, and progressive fat accumulation in different body regions. Abdominal fat induces oxidative stress, which in turn has been shown to cause low-level chronic systemic inflammation [50, 51]. In OVX rats, weight gain and excess of visceral adipose tissue are well-described phenotypic changes caused by this model [35, 52, 53]. In contrast to the previously described effects of curcumin in inhibiting weight gain in high-fat diet models [54, 55], our results showed that it did not reverse the weight gain caused by OVX. Perhaps the 30-day period of treatment with curcumin has been sufficient to only exert direct effects on the intestinal accumulated fat but not in overweight, since the studies above mentioned used curcumin at larger amounts for longer periods (12 and 24 weeks) [55, 56]. In addition, these studies looked at the effects in male rodents, which could be a considerable difference, given that the presence/absence of female sex hormones may be decisive for the imbalance in weight gain [57]. Indeed, more studies are claimed to determine the possible intervention of curcumin on the overweight caused by OVX in rats.

Estrogen is a key modulator of lipid homeostasis; consequently postmenopausal women usually exhibit increased levels of LDL and total cholesterol and decreased HDL, compared to premenopausal [58]. In OVX models, some alterations in lipid profile were already described such as increases in LDL or non-HDL and total cholesterol levels [28, 53]. In our model, OVX* per se* increased both LDL and total cholesterol, but no alterations in HDL fraction were found. Otherwise, curcumin intake seemed to improve lipid profiles of OVX rats by keeping the level of these lipids similar to sham group, or even by decreasing TG and VLDL levels irrespective of the noneffect of OVX upon these parameters. In experimental models of atherosclerosis in rabbits, curcumin showed antioxidant effects on LDL [59]. In addition, curcumin was also able to decrease the concentrations of oxidized HDL and LDL in the serum of women ranging from 40 to 90 years old without any toxic effect on the hepatic and renal tissues [60]. It has been proposed that curcumin and its metabolites function as peroxisome proliferator-activated receptor (PPAR*γ*) ligands, thus explaining its action as a hypolipidemic agent [61].

Increased secretion of inflammatory cytokines is thought to be involved in pathogenesis of numerous aging diseases, and clinical trials strongly support a link between increased levels of TNF-*α* and IL-6 with menopause-related bone loss and cardiovascular diseases [62–64]. Furthermore, excess of adiposity is associated with greater systemic inflammation [51]. Using the OVX model, Wang et al. 2012 [65] did observe that estrogen would be protective against hepatocellular metastasis by reducing IL-6 levels. In addition, Kireev et al. 2010 showed that ovariectomy is associated with an increase in proinflammatory cytokines (TNF-*α*, IL-1*β*, and IL-6) and reduction of the anti-inflammatory IL-10, leading to increases in lipoperoxidation in liver tissues [39]. It has been described that curcumin may exert inhibitory actions on the levels of the proinflammatory cytokines TNF-*α* and IL-6, thus reducing inflammation and oxidative damage induced by cadmium intoxication [66]. In our model, IL-6 levels were slightly increased in serum samples of OVX rats and curcumin treatment could restore the IL-6 levels to sham values at higher doses. In fact, curcumin is a well-known inhibitor of transcription factors involved in cytokine production as NFkappaB and STAT-3 [67, 68], which could be underlying its anti-inflammatory action in different disease models. Last, OVX rats are described to present increases in ALT and AST levels with 21 days after surgery [69]. In our model (90 days after surgery), although ALT was not altered, the serum levels of aspartate aminotransferase (AST) not surprisingly were slightly increased by experimental menopause, suggesting the presence of chronic low-level tissue damage; curcumin was unable to rescue the normal patterns. Based on all aforementioned effects of curcumin upon oxidative, lipid, and inflammatory parameters herein and previously described in different preclinical and clinical models, one therefore could conclude that curcumin harbors some potential for decreasing the predisposition to cardiovascular disease and other complications associated with menopause and ageing.

Taken together, we showed that curcumin was able to minimize the alterations in IAT accumulation, IL-6 serum levels, lipid profile, and oxidative stress caused by sexual female hormone deprivation in OVX rats. We also observed that the nonenzymatic antioxidant potential of curcumin seems to be a key component of this response, at least regarding the oxidative stress parameters. Our results and previous work from other groups [2, 17, 19, 70, 71] collectively support a more in-depth clinical trial investigation on the curcumin potential and safety to treat menopausal patients aiming to ameliorate aging-related processes frequently accelerated by menopausal status.

## Figures and Tables

**Table 1:** Body weight gain, individual uterus, and visceral adipose tissue weight (g) in relation to respective animal body weight (g).

	Sham	OVX	Curcumin (mg/Kg/day)	
			OVX 50	OVX 100
Weight gain (g)	22.62 ± 2.618	61.818 ± 3.60^d^	59 ± 3.37^c^	61.625 ± 2.77^d^
Uterus (g)/body mass (g) ratio	1 ± 0.07	0.17 ± 0.01^d^	0.18 ± 0.01^d^	0.19 ± 0.02^d^
Visceral adipose tissue (g)/body mass (g) ratio	1 ± 0.05	1.41 ± 0.1^c^	0.99 ± 0.04^#^	1.08 ± 0.09^*∗*^

Fresh uterine and visceral adipose tissues weight from Sham and OVX rats. Data are shown as mean ± SEM (sham *n* = 8, OVX *n* = 11, OVX50 *n* = 8, and OVX100 *n* = 8).

^c^ *P* < 0.01: different from sham group; ^d^ *P* < 0.001: different from sham group; ^**∗**^ *P* < 0.05: different from OVX group; ^#^ *P* < 0.01: different from OVX group (one-way ANOVA followed by Tukey's test).

OVX: ovariectomized.

**Table 2:** Serum parameters and lipid profile.

	Sham	OVX	Curcumin (mg/Kg/day)	
			OVX 50	OVX 100
IL-6 (pg/mL)	74.73 ± 1.08	78.80 ± 0.65^c^	75.96 ± 0.56^**∗**^	75.39 ± 0.72^**∗**^
AST activity (U/dL)	18.937 ± 1.72	26.888 ± 1.37^c^	25.507 ± 1.22^b^	26.975 ± 0.98^c^
ALT activity (U/dL)	16.974 ± 0.67	16.753 ± 0.68	16.739 ± 0.48	16.979 ± 0.91
HDL (mg/dL)	30.714 ± 1.99	31.666 ± 1.55	31.285 ± 1.12	29.857 ± 1.47
LDL (mg/dL)	17.285 ± 2.74	26.933 ± 3.40^b^	26.171 ± 1.36	23.742 ± 1.46
VLDL (mg/dL)	9.428 ± 1.19	8.222 ± 0.75	5.714 ± 0.42^b^	6.285 ± 0.86^b^
Total cholesterol (mg/dL)	54.750 ± 3.56	70.888 ± 4.41^b^	63.857 ± 2.67	59.714 ± 3.04
Total triglycerides (mg/dL)	47.142 ± 5.88	40.888 ± 2.27	27.714 ± 2.07^d^	30.571 ± 4.41^d^

IL-6 levels, AST/ALT activities, and lipid profile were measured in serum samples. Sham and OVX groups were treated once a day for 30 days with refined olive oil containing or not curcumin. Data are expressed as mean ± SEM (sham *n* = 8, OVX *n* = 11, OVX50 *n* = 8, OVX100 *n* = 8).

Statistically different from Sham group: ^b^ *P* < 0.05, ^c^ *P* < 0.01 ^d^ *P* < 0.001; ^**∗**^ *P* < 0.05: different from OVX group (one-way ANOVA followed by Tukey's test).

ALT: alanine aminotransferase; AST: aspartate aminotransferase; HDL: high-density lipoprotein; IL-6: interleukin-6; LDL: low-density lipoprotein; OVX: ovariectomized; VLDL: very low-density lipoprotein.

**Table 3:** 

Time of induction 50% (seconds)		
Treatments	Serum	Liver
Sham	1380	715
OVX	780	450
OVX + 50 mg/kg	961	603
OVX + 100 mg/kg	1260	665

## Abbreviations

AAPH: 2,2-Azobis[2-amidinopropane]
ANOVA: Analysis of variance
AUC: Area under curve
CAT: Catalase
DNPH: Dinitrophenylhydrazine
DTNB: 5,5-Dithionitrobis 2-nitrobenzoic acid
GPx: Glutathione peroxidase
HDL: High-density lipoprotein
HRT: Hormone replacement therapy
IAT: Intestinal adiposity accumulation
LDL: Low-density lipoprotein
OVX: Ovariectomized
ROS: Reactive oxygen species
TRAP: Total reactive antioxidant potential
SOD: Superoxide dismutase
TBARS: Thiobarbituric acid reactive species
VLDL: Very low-density lipoprotein.
